# Aripiprazole-induced sleep-related eating disorder: a case report

**DOI:** 10.1186/s13256-018-1622-2

**Published:** 2018-04-05

**Authors:** Nobuyuki Kobayashi, Masahiro Takano

**Affiliations:** 1 Department of Psychosomatic Medicine, Coloproctology Center Takano Hospital, 3-2-55 Oe Chuou-ku, Kumamoto, 862-0971 Japan; 2 Coloproctology Center Takano Hospital, 3-2-55 Oe, Chuou-ku, Kumamoto, 862-0971 Japan

**Keywords:** Adjunctive therapy, Aripiprazole, Atypical antipsychotics, Side effect, Sleep-related eating disorder

## Abstract

**Background:**

Sleep-related eating disorder is characterized by parasomnia with recurrent episodes of nocturnal eating or drinking during the main sleep period. Several drugs, including atypical antipsychotics, induce sleep-related eating disorder. However, aripiprazole has not previously been associated with sleep-related eating disorder.

**Case presentation:**

A 41-year-old Japanese man visited our clinic complaining of depression. The patient was treated with sertraline, which was titrated up to 100 mg for 4 weeks. A sleep inducer and an anxiolytic were coadministered. His depressive mood slightly improved, but it continued for an additional 4 months. Subsequently, aripiprazole (3 mg) was added as an adjunctive therapy. After 3 weeks, the patient’s mother found that the patient woke up and ate food at night. The next morning, the patient was amnesic for this event, felt full, and wondered why the bags of food were empty. This episode lasted for 2 days. The patient gained 5 kg during these 3 weeks. After the aripiprazole dose was reduced to 1.5 mg, the patient’s nocturnal eating episodes rapidly and completely disappeared.

**Conclusions:**

To the best of our knowledge, this is first report of sleep-related eating disorder induced by aripiprazole, and it indicates that this disorder should be considered a possible side effect of aripiprazole. Although aripiprazole is used mainly in patients with schizophrenia, its recently documented use as an adjunctive therapy in patients with depression might induce hitherto unknown side effects.

## Background

Sleep-related eating disorder (SRED) is characterized by parasomnia with recurrent episodes of night eating or drinking during the main sleep period. Affected individuals have one or more features, including consumption of peculiar foods, insomnia related to sleep disruption, sleep-related injury, dangerous behaviors performed while in pursuit of food or cooking food, morning anorexia, and adverse health consequences. Individuals with SRED typically have full or almost complete amnesia for these events [1]. SRED is thought to be a sleep disorder and is distinct from night eating syndrome [2]. Patients with night eating syndrome have episodes of waking from nocturnal sleep and eating at least one-third of their daily calories. They are aware of their night arousal and hyperphagia.

SRED diagnostic criteria do not include the induction of this condition by medication or a physical condition [1]. However, several medications have been noted to induce SRED-like behavior. Benzodiazepine receptor agonists are the most potent inducers of SRED [3]; zolpidem is often reported to be the cause of medication-associated SRED [4–6]. Atypical antipsychotics, such as olanzapine [7], risperidone [8], and quetiapine [9, 10], are also known to induce SRED.

Aripiprazole is an atypical antipsychotic that is widely used for the treatment of schizophrenia, bipolar disorder, and depression [11]. For the treatment of depression, only adjunctive therapy with aripiprazole is indicated. Generally, after selective serotonin reuptake inhibitor (SSRI) or serotonin-noradrenaline reuptake inhibitor (SNRI) treatment is ineffective, a combination of SSRI/SNRI and aripiprazole is used [11]. In our clinic, we noted a patient with depression who exhibited SRED after aripiprazole was introduced as an adjunctive therapy. To the best of our knowledge, this is the first report of a patient with SRED associated with aripiprazole.

## Case presentation

A 41-year-old Japanese man with depression complained of nighttime eating. The patient could not remember these events the next morning. He has been obese for 20 years. He had no history of sleep apnea, restless leg syndrome, somnambulism, or eating disorders and no symptoms similar to binge eating or night eating. He had no family history of any sleep-related disorders. He had developed depression and insomnia because of stress after 5 months on a new job, and he visited our clinic 2 months later. He was not receiving any medication and did not take herbal medicine or supplements. He did not have suicidal thoughts or self-harm behavior. During his first visit, a physical examination revealed that his height was 167 cm, body weight was 90 kg, and body mass index (BMI) was 32.2 kg/m^2^. His laboratory test results revealed slight liver damage and hyperlipidemia, with a serum alanine aminotransferase level of 52 U/L (normal range, 10–42 U/L), aspartate aminotransferase level of 35 U/L (normal range, 13–30 U/L), and triglyceride level of 277 mg/dl (normal range, 40–150 mg/dl). His Self-Rating Depression Scale score was 62 (normal range, 27–47; range of values, 20–80) [12].

The patient was diagnosed with depression and obesity. Along with supportive psychotherapy, he began sertraline treatment prior to sleep; his dose was titrated up to 100 mg (Japanese full dose) over 4 weeks. In addition, he received the sleep inducers zolpidem and flunitrazepam, and the anxiolytic clotiazepam. His depressive mood slightly improved, but it continued for an additional 4 months despite resolution of insomnia. Subsequently, 3 mg of aripiprazole was added as an adjunctive therapy. His depressive mood immediately improved, and his appetite increased. The patient reported enjoying driving and cleaning up his room, but felt fatigued the next morning. Three weeks after the initiation of aripiprazole treatment, the patient’s mother found that the patient woke up and ate food from the refrigerator during the night. The patient did not respond to the mother during this episode. The patient did not remember this event, felt full, and wondered why the bags of food were empty the next morning. He did not consume any improper substances such as raw, frozen, or spoiled food. This episode lasted for 2 days. During the 3 weeks after aripiprazole treatment, the patient gained 5 kg. The patient acknowledged that he remembered urinating at night only once before starting aripiprazole. Re-examination of laboratory results revealed that the patient’s serum glucose, cortisol, and thyroid hormone levels were normal. He was advised to reduce his aripiprazole and zolpidem doses. However, he continued to use the same zolpidem dose because his insomnia returned after a dose reduction.

**Fig. 1:**
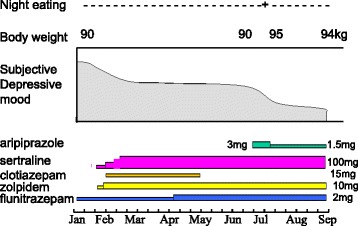
Clinical course. The addition of aripiprazole induced night eating, which was diminished by a dose reduction of aripiprazole. Night eating: + = present, − = absent

After aripiprazole was reduced to 1.5 mg, the patient’s nocturnal eating episodes rapidly and completely disappeared. However, amnesia of nocturnal urination occurred three times during the next 6 months. This amnesia of nocturnal urination disappeared after zolpidem was replaced with triazolam. The patient’s depressive mood continued to improve, and his body weight remained at 94 kg (Fig. 1). He did not exhibit any psychotic features during his entire treatment.

## Discussion

In this case study of a patient with depression, the addition of aripiprazole to SSRI treatment induced an immediate subjective improvement in mood, weight gain, and SRED. SRED diminished shortly after reduction of the aripiprazole dose. No other medication was changed during this period. This time course strongly suggests that SRED in our patient was induced by aripiprazole. Common side effects of atypical antipsychotics such as aripiprazole include akathisia, diabetes mellitus, and weight gain [13]. Among the atypical antipsychotics, aripiprazole is generally thought to have a reduced likelihood of inducing weight gain [14]; however, aripiprazole can cause weight gain when coadministered with an SSRI or SNRI as adjunctive therapy [15]. This effect may have been more marked in the patient described in this case report, and drug-induced hyperphagia may have played a role in the development of SRED.

Several medications have been reported to induce SRED [3]. Anxiolytics and sedatives, such as the benzodiazepine agonist zolpidem, are the most frequently reported [4–6]. Our patient also received zolpidem, which has no apparent correlation with SRED but may induce amnesia of nocturnal urination. Thus, zolpidem may have played a role in aripiprazole-induced SRED.

Table 1 shows the reported SRED cases associated with atypical antipsychotics. It includes one, one, and three cases of SRED induced by olanzapine [7], risperidone [8], and quetiapine [9, 10], respectively, and also includes our patient with SRED, in whom we believe SRED was induced by aripiprazole. It is of interest that the target disease for these antipsychotics was mood disorder in five of the six cases and did not include schizophrenia, the main indication for atypical antipsychotics. Relatively low doses of antipsychotics induced SRED in the reported cases. Racial differences among the cases were not remarkable: two patients were Asian and two were African American. Obesity was seen in three of the four patients whose BMI was provided in the case reports. Therefore, induction of SRED by atypical antipsychotics may be influenced by the patient’s metabolic background/predisposition (along with environmental conditions/exposures).

**Table 1:** Reported cases of sleep-related eating disorder induced by atypical antipsychotics

Subject no.	Age	Sex	Drug name	Dose (mg)	Target disease	Race	BMI (kg/m^2^)	Reference
1	52	M	Olanzapine	10	Bipolar I	Belgium^a^	nd	[7]
2	68	M	Risperidone	2	Psychotic disease due to vascular dementia	Taiwan^a^	nd	[8]
3	51	M	Quetiapine	150	Depression	African American	34.1	[9]
4	50	F	Quetiapine	200	Depression	African American	30.5	[9]
5	48	F	Quetiapine	100	Bipolar disorder	United States^a^	25.6	[10]
6	41	M	Aripiprazole	3	Depression	Japanese	32.2	Present report

*Abbreviations: BMI* Body mass index, *nd* Not described

^a^not described, but the nation of the report authors’ affiliation is provided

Atypical antipsychotics act as dopaminergic antagonists or stabilizers and can possess serotonergic activities [11, 16]. Some researchers have reported resolution of SRED cases by the use of dopaminergic agonists [17, 18]. One possible explanation is that the SRED seen in our patient was induced by the dopaminergic antagonistic action of aripiprazole. Although aripiprazole was reported to have relatively minor effects on sleep among atypical antipsychotics [19], authors of one report found that aripiprazole was effective for treatment of delayed sleep phase syndrome [20]. Aripiprazole might thus play some role in sleep–wake interactions. It is possible that hitherto unknown side effects of aripiprazole might develop in adjunctive therapy for depression, because it is uncertain how aripiprazole acts as an adjunctive therapy [13]. Further research is needed to clarify its pathogenesis.

To date, there are no reports of aripiprazole-induced SRED. However, the results of our present case study indicate that SRED should be considered as a possible side effect of aripiprazole.

## Conclusions

To the best of our knowledge, this is first report of SRED induced by aripiprazole. SRED should be considered a possible complication of aripiprazole. This side effect might be caused by the use of aripiprazole as adjunctive therapy for depression.
